# Mental Health Among Spanish Adults During the COVID-19 Pandemic and in the Post-Pandemic Period: A Gender Analysis

**DOI:** 10.3390/medicina61101734

**Published:** 2025-09-24

**Authors:** M. Pilar Matud, Lorena Medina, Ignacio Ibañez, Maria-José del Pino

**Affiliations:** 1 Department of Clinical Psychology, Psychobiology, and Methodology, Universidad de La Laguna, 38200 San Cristobal de La Laguna, Spain; lorenamr@gmail.com (L.M.); iibanez@ull.edu.es (I.I.); 2 Department of Sociology, Universidad Pablo de Olavide, 41013 Sevilla, Spain; mjpinesp@upo.es

**Keywords:** COVID-19, mental health, gender, distress, well-being, self-esteem, resilience, social support, stressful events, adulthood

## Abstract

*Background and Objectives*: The COVID-19 pandemic has had severe and far-reaching repercussions for health systems and societies, creating a global mental health crisis that has persisted after the pandemic. This study examined differences in mental health (i.e., psychological distress and well-being) by gender and pandemic period (i.e., lockdown, second and third pandemic waves, the last year of the pandemic, and the post-pandemic period) among Spanish individuals in two stages of adulthood: established adulthood and midlife. The second objective was to identify the risk and protective factors for psychological distress and well-being during the post-pandemic period. *Materials and Methods:* The study design was repeated cross-sectionally. The non-probability sample included 3677 people (66.2% women) from the general Spanish population, aged 30 to 59 years. Participants were assessed between 1 June 2020 and 23 May 2024 using the following self-reports: the General Health Questionnaire-12 item version, the Scale of Positive and Negative Experience, the Brief Inventory of Thriving, the Satisfaction with Life Scale, the Rosenberg Self-Esteem Scale, the Social Support Scale, the Stressful Events Questionnaire, the Brief Resilience Scale, and the Perceived Vulnerability to Disease Questionnaire. *Results*: Although rates of psychological distress varied across pandemic periods, women consistently experienced higher rates than men. During the post-pandemic period, the rates of psychological distress were 47.5% for women in established adulthood and 34.7% for midlife women, compared to 28.6% and 22% for men, respectively. Low self-esteem was the main predictor of greater psychological distress and lower well-being for both genders and age groups in the post-pandemic period. Another significant predictor was lower stress resilience. *Conclusions*: The COVID-19 pandemic threatened women’s mental health and well-being more than men’s, and this threat persists after the pandemic, especially among women in established adulthood. The study’s findings are relevant for the design of policies, programs, and strategies to achieve better mental health and well-being among citizens and greater gender equality.

## 1. Introduction

Coronavirus disease 2019 (COVID-19) defines the symptomatic disease caused by infection with severe acute respiratory syndrome coronavirus 2 (SARS-CoV-2) [1]. On 11 March 2020, the World Health Organization (WHO) characterized it as a pandemic, prompting countries to take urgent and aggressive action [2]. According to the WHO, the COVID-19 pandemic, one of the greatest global crises in generations, has had serious and far-reaching consequences for health systems, societies, and economies [3]. The fear and uncertainty surrounding the pandemic, together with the social restrictions imposed to control the virus’s spread, impacted everyone’s daily life [4,5,6]. Measures such as lockdowns, school closures, self-isolation, border closures, travel restrictions, and social distancing not only impacted personal and social lives but also had significant economic consequences for all countries, intensifying economic challenges [4,7,8] and affecting employment [4,9]. While the management of the COVID-19 pandemic and resulting outcomes varied widely between countries, with national variations in the implementation of public health fundamentals such as isolating infected individuals, lockdowns, quarantines, and social and financial support for people in isolation or quarantine [10], the COVID-19 pandemic has had a devastating impact on people worldwide [3,6,11], and has created a global mental health crisis [12]. This global mental health crisis has persisted after the pandemic [11]. Demand for mental health services has reached an unprecedented level, overwhelming services and lengthening waiting lists [13].

### 1.1. Theoretical Rationale

The COVID-19 pandemic posed one of the largest collective challenges of our lifetime [6]. This pandemic and the public health measures issued in response have imposed various stressors on individuals, including contracting COVID-19 or having close relatives down with COVID-19, concerns about the safety of others in their care, and the stress of living in the pandemic [6]. These stressors may accumulate to undermine mental health [6]. According to the WHO, among its many impacts, the pandemic has created a global mental health crisis, fueling short- and long-term stresses and undermining the mental health of many people [12]. Mental health “is an integral part of our general health and well-being and a basic human right. Having good mental health means we are better able to connect, function, cope and thrive” [12] (p. xiv). Although many studies have been conducted on the effects of the pandemic on mental health, most of these studies were conducted during the early stages of the pandemic or during the first year. Many studies focused on the mental health of the population during lockdown. Lockdown has been defined as “an emergency and temporary measure imposed by governmental authorities that (1) applies to a city, region, or nation to prevent the spread of the COVID-19 virus; (2) is mandatory and applied indiscriminately to a general population; and (3) requires citizens to stay at home and refrain from or limit social and economic activities outside the home” [14] (p. 8). Some studies have found elevated mental health symptoms and stress during lockdown [15,16,17,18,19], but the results are heterogeneous. It has been found that the effects vary depending on other variables, including gender, age, marital status, occupation, and perceived vulnerability to infection. The effects have been greater in women [15,17,18,19], students [17], and people with higher perceived vulnerability to infection [17], while older age has been shown to decrease the likelihood of experiencing mental health symptoms [15,19]. Because many of these studies were cross-sectional, it has been suggested that although lockdowns to control the spread of the virus have had important impacts on daily life, their effects on mental health are unclear [14]. A longitudinal study conducted in Austria during the COVID-19 lockdown and six months later found that the detrimental health consequences of the pandemic persisted several months after its outbreak and the end of the lockdown, suggesting that the COVID-19 pandemic caused long-lasting detrimental effects on mental health [16]. However, a review and meta-analysis study of longitudinal studies and natural experiments investigating the relationship between COVID-19 lockdowns and mental health found that lockdowns had small effect sizes for depression and anxiety, whereas those for loneliness, general distress, negative affect, suicide risk, and social support were not significant. This review and meta-analysis, which included 25 studies involving 72,004 participants and 58 effect sizes, indicated important heterogeneity among the studies. The findings suggest that the psychological impact of COVID-19 lockdowns was small in magnitude and highly heterogeneous, indicating that lockdowns do not uniformly negatively impact mental health and that most individuals are psychologically resilient to their effects [14].

Despite significant heterogeneity across studies [5,14,20], there is evidence that the prevalence of mental health problems has increased since the outbreak of the COVID-19 pandemic [3,6,11,21,22]. A systematic review and meta-analysis of studies from 1 January 2020 to 11 July 2020, which collected information from 32 different countries and 398,771 participants, found that while the prevalence of mental health issues varied widely across countries and regions, it was higher than before the COVID-19 outbreak began. The estimated global prevalence was 50.0% for psychological distress, 36.5% for stress, 28.0% for depression, 27.6% for sleep problems, 26.9% for anxiety, and 24.1% for post-traumatic stress symptoms [23]. Although heterogeneity also prevailed, some longitudinal studies found that compared to pre-pandemic levels, there was an overall increase in mental health symptoms but subsequently a decrease [24,25,26,27], though sampling predominantly European and North American adults and changes depended on the symptom assessed [24]. Signs of strain were also shown by some components of subjective well-being, such as the increase in negative emotions. However, others, such as life satisfaction, revealed notable signs of resilience [6].

Some groups were affected much more than others [3,6,22]. The prevalence of mental health issues during the pandemic varied widely across regions and countries [23], as well as according to sociodemographic and personal characteristics. This variation suggests the existence of risk and protective factors for mental health. While no consensus has been reached, these factors include having pre-existing health risks, sociodemographic factors such as gender, age, occupation, marital status, living in urban vs. rural areas, and belonging to a minority or marginalized racial group, as well as psychosocial factors such as social support, resilience, and self-esteem. The role of factors, such as family composition and relationship status, has also been suggested as the pandemic has substantially altered daily life [6]. Social determinants of mental health and social risk factors, such as institutional racism, socioeconomic status, sociopolitical status, age, gender, and urbanity/rurality, have also been raised as risk factors that may interact with pandemic stressors to increase the risk of negative mental health outcomes [22]. Clinical vulnerabilities, including pre-existing physical and/or mental health conditions, increase the risk of negative outcomes, especially distress and anxiety. Physical conditions, such as chronic obstructive pulmonary disease (COPD) and diabetes, as well as treatments such as immunosuppressants, which increase the risk of severe infection, likely increase the risk of negative mental health outcomes [22]. Studies have generally found a higher risk of mental health problems in women compared to men [5,6,20,28,29,30,31,32], in younger people compared to older people [5,11,28,29,30,31,32], in students [5,29,33], and in people living without a partner compared to those married or living with a partner [6,19,32,34,35]. High social support [35,36,37,38,39], high resilience [31,32,37,40], and high self-esteem [39,40,41,42] are among the protective psychosocial factors.

It has been suggested that the pandemic is likely to exacerbate existing gender inequalities that favor men over women, placing women at a greater risk of distress, depression, and traumatic stress [22]. Gender differences in mental disorders are found consistently in epidemiological surveys. Women had more anxiety–mood disorders than men and men more externalizing substance disorders than women [43]. Gender refers to the different socially constructed roles, norms, activities, behaviors and attributes that a given society considers appropriate for men and women [44]. It is a complex social system that structures how all human beings live, and gender norms support a hierarchy of power and privilege that favors what is considered male or masculine over what is considered to be female or feminine. This reinforces systemic gender inequality. These inequities result in health inequities [45]. According to Heise et al., “Gender inequality and restrictive gender norms are powerful but separate determinants of health and wellbeing” [45] (p. 2440). Although gender differences in mental health have been found generally consistent across cohorts, significant narrowing was found in recent cohorts for major depressive disorder and substance disorders [43]. This suggests that gender differences in mental health are a dynamic process. It has also been suggested that sex interacts with age and other social variables to generate different health and well-being outcomes. In a recently published study on emotion recognition—a pivotal component of social functioning and psychological well-being—gender-specific patterns were found in the relationship between aging and emotion recognition [46].

The high risk of mental health problems during the COVID-19 pandemic is thought to be related to several psychological and social pathways that may interact over the life course [47]. Therefore, it is important to conduct studies focusing on specific life periods. In this study, two life stages of the adult period are examined: established adulthood (ages 30 to 44) and midlife (ages 45 to 59). During these life stages, work and family are the two main areas in all societies [48]. These areas have been significantly affected by the COVID-19 pandemic, especially during the outbreak and first year of pandemic and lockdown periods. It has been suggested that in developed countries, the years between 30 and 44 are, for many people, the most demanding, intense, and reinforcing years of adult life [48]. During this stage of life, most people face multiple demands: becoming experts in their field, advancing their job status and achievements, maintaining relationships, and caring for young children [48]. Established adulthood differs from midlife in terms of career, with most people in midlife having reached the peak of their career; in terms of family, with people in midlife generally having older children; and in terms of health, with people in midlife being more likely to experience age-related changes such as menopause for women and andropause for men [48].

These are not homogeneous groups. Differences based on variables such as economic status, occupation, education level, marital status, and number and age of children are important. However, these are two particularly demanding stages of women’s lives. They are generally expected to be responsible for family care and domestic tasks. In addition, they are generally more disadvantaged in the labor market than that of men [49,50,51]. Women and girls constitute half of the world’s population, and thus, half of the world human potential. However, gender inequality persists everywhere. Sexual violence and exploitation, the unequal division of unpaid care and domestic work, and discrimination in public office all remain significant barriers [49]. On average, women occupy only 30 percent of managerial positions in the labor market globally [49] and earn 23 percent less than men globally [50]. Furthermore, women spend more time on unpaid domestic and care work than men do [49,50,51]. They bear primary responsibility for childcare and are twice as likely as men to spend at least five hours per day on childcare. This unequal distribution of childcare responsibilities creates significant obstacles for women participating in the labor market on an equal footing with men. Caregiving responsibilities significantly impact work–life balance, resulting in fewer working hours, limited career progression, and financial consequences. This especially affects mothers of young children [51]. These variables are relevant to gender differences in health and well-being. A study on time use, health, and well-being throughout the life cycle conducted in Spain [52] found statistically significant differences in how women and men use their time. Women spent more time than men on housework, childcare, and caring for sick relatives. In contrast, men spent more time than women on enjoying activities and exercising. Generally, there were no age-based differences in time use among men. However, the time women spent on housework increased with age, while the time they spent on physical exercise decreased. Additionally, midlife women spent the most time each day caring for sick relatives, whereas women in established adulthood spent the least time on enjoyable activities. For both genders, more time spent exercising was associated with better health and well-being. However, more time spent on housework was associated with poorer health and well-being in women [52].

The mortality rate was very high in the early stages of the COVID-19 pandemic due to the novelty of the disease, for which there was no vaccine or previous immunity [53,54]. However, advances in treatment and vaccines led to a change in the situation. By the end of 2021, 58% of the global population had received some vaccine and probably 73–81% of the global population had been infected, vaccinated, or both [55]. Death rates were reduced due to the administration of more than 12.8 billion vaccine doses globally and the decreased pathogenicity of new SARS-CoV-2 variants [56]. On 5 May 2023, the WHO [57] determined, given the decline in COVID-19 hospitalizations and intensive care unit admissions, as well as the population’s high level of immunity to SARS-CoV-2, “that COVID-19 is now an established and ongoing health issue which no longer constitutes a public health emergency of international concern”. Although it was considered time to transition to long-term management of the COVID-19 pandemic, it was recognized that uncertainties regarding the possible evolution of SARS-CoV-2 remain [49] and the COVID-19 was still considered a global threat [58].

### 1.2. COVID-19 Pandemic in Spain

The first case of the novel coronavirus (SARS-CoV-2) in Spain was diagnosed on 31 January 2020, and this coronavirus spread rapidly throughout the country. The first pandemic wave started in mid-March 2020 and lasted three months [59]. On 15 March 2020, the Spanish government imposed a national lockdown involving stringent measures and declared a state of emergency under Royal Decree 463/2020, which was extended until 21 June 2020. This lockdown, which was imposed to limit viral transmission and to avoid overwhelming the healthcare system [54], had a profound impact on daily life, employment, and the Spanish economy [59]. The strict lockdown and home confinement abated the first wave of the pandemic. However, relaxing these measures during the summer of 2020 led to a second wave of the pandemic from mid-September to the end of the year [54,59]. This was followed by a third wave from early 2021 to March 2021, which resulted in the highest peak of cases, although the number of deaths was much lower than during the first pandemic wave [54]. On 25 October 2020, the Spanish government declared a new state of alarm to contain the spread of SARS-CoV-2 infections. Widespread measures, such as social distancing and limited gatherings, were implemented alongside stricter, more localized measures. Spain has a decentralized healthcare system that allowed each of its 17 regions or autonomous communities to impose their own limitations [54]. Consequently, educational centers and businesses were no longer closed nationwide.

In 2020, Spain was one of the European countries most affected by the COVID-19 pandemic, with approximately 80,000 excess deaths that year [59]. Madrid, the capital of Spain, was the epicenter of the COVID-19 pandemic in the country [59,60], primarily due to its high population density and mobility. By Easter 2021, approximately 25% of Madrid’s population had been infected with SARS-CoV-2, and up to 15 March 2021, 14,000 people had died in the Madrid region [60]. Though there were regional variations in infection rates, the initial pandemic containment measures profoundly impacted daily life, employment, and the Spanish economy. Since the pandemic was declared on 11 March 2020 [2], European Union member states have been forced to close their borders, establish mobility restrictions, and implement social distancing rules to contain the spread of the virus. These measures have strongly impacted the tourism industry, an important sector of Spain’s GDP [61]. Between the onset of the pandemic and early 2022, Spain experienced six waves, though the clinical severity of the infection and number of deaths decreased significantly compared to the first wave [54,59]. Vaccination of the Spanish population began in January 2021, and the third dose was administered in September of that year [54]. During the last year of the pandemic, most of the Spanish population was vaccinated, the disease was not serious for most people, and there were virtually no social restrictions due to the COVID-19 pandemic, except for mask mandates in healthcare and social care centers and on public transportation.

The main aim of this work is to determine whether there are differences in mental health (psychological distress, stress, social support, and well-being) by gender and pandemic period (lockdown, second and third pandemic waves, last year of the pandemic and post-pandemic) among Spanish individuals in two stages of adulthood: established adulthood (between 30 and 44 years) and midlife (between 45 and 59 years). A second aim is to identify risk and protective factors for psychological distress and well-being during the post-pandemic period among women and men in the two life stages studied.

## 2. Materials and Methods

### 2.1. Study Design and Participants

The study design is cross-sectionally repeated with a non-probability sample consisting of 3677 people (66.2% women) from the general Spanish population, aged between 30 and 59 years, who were assessed using self-report measures. Their main demographic characteristics are shown in Table 1. As can be seen, there were no differences between women and men in age or number of children. However, there were differences in the remaining sociodemographic characteristics. Although there were varying levels of education, it was less common to have only a basic education and more common to have a university education, which was more common for women (50.6%) than for men (42.0%). Most participants were employed, 88.9% of men and 78.6% of women, and women (15.7%) were more likely to be unemployed than men (8.4%). More than half of the participants (66.6% of the men and 63.3% of the women) were married or living with their partner. Almost one fifth had never been married and 12.1% of men and 16.0% of women were separated, divorced, or widowed.

All participants were assessed during four phases of the COVID-19 pandemic: (1) During the Spanish national lockdown, between 1 June and 18 June 2020 (*n* = 544). (2) During the second and third waves in Spain, between 15 October 2020 and 2 March 2021 (*n* = 1104). (3) During the last year of the pandemic, between 9 February 2022 and 28 February 2023 (*n* = 954). (4) During the post-pandemic period, between 2 October 2023 and 23 May 2024 (*n* = 1075).

### 2.2. Instruments

All participants completed the following instruments:

- General Health Questionnaire, 12-item version (GHQ-12) [62]. The GHQ-12 is a widely used tool in epidemiological research and a popular measure of psychological distress [63]. It consists of 12 items with several scoring options, two of which were used in this study: the Likert method (0-1-2-3) and the GHQ scoring method (0-0-1-1). The Likert score was used in all analyses, except for discriminating cases with distress from cases without distress, for which the GHQ score was used. According to Lundin et al. [64], the most appropriate threshold for distinguishing cases with distress from cases without distress is ≥ 4. This threshold has a sensitivity of 81.7 and a specificity of 85.4. For the current sample, Cronbach’s alpha was 0.90 when the 12 items were scored using the Likert method and 0.89 when scored using the GHQ scoring method.
- Scale of Positive and Negative Experience (SPANE) [65]. The SPANE is a 12-item scale in which 6 items assess positive feelings, and 6 items assess negative feelings. Each item is scored on a 5-point scale ranging from 1 (very rarely or never) to 5 (very often or always). The positive and negative scales are scored separately and then combined to measure affect balance. Higher scores indicate that positive feelings are experienced more than negative feelings. For the current sample, Cronbach’s alphas were 0.93 for positive feelings, 0.87 for negative feelings, and 0.92 for affect balance.
- Brief Inventory of Thriving (BIT) [66]. The term “Thriving denotes the state of positive functioning at its fullest range—mentally, physically, and socially” and the BIT has been designed to measure the core of psychological well-being [66] (p. 256). The scale consists of 10 items with a 5-point Likert response scale ranging from 1 (strongly disagree) to 5 (strongly agree). Cronbach’s alpha was 0.90 for the current sample.
- Satisfaction with Life Scale (SWLS) [67]. The SWLS is a scale designed to measure global life satisfaction as a cognitive–judgmental process [67]. It consists of 5 items with a 7-point response scale ranging from 1 (strongly disagree) to 7 (strongly agree). Cronbach’s alpha for the current sample was 0.89.
- Rosenberg Self-Esteem Scale (RSES) [68]. The RSES is a 10-item scale that measures self-esteem and is the most widely used measure of global self-esteem [69]. The response scale is 4-point. It ranges from 0 (strongly disagree) to 3 (strongly agree). In the present study sample, Cronbach’s alpha was 0.85.
- Social Support Scale (SSS) [70]. This 12-item scale assesses perceived social support and is structured into two factors: emotional social support (7 items) and instrumental social support (5 items). The response scale has five points, ranging from 0 (never) to 3 (always). For the current sample, Cronbach’s alpha was 0.91 for the emotional social support factor and 0.89 for the instrumental social support factor.
- Stressful Events [39]. Participants were asked if, since the onset of the COVID-19 pandemic, they had experienced any of the following events and/or losses: (1) loss of employment, (2) financial problems, (3) major disagreement with partner, (4) major disagreement with family, (5) illness of family members or loved ones, (6) death of one or more family members or loved ones, (7) own illness, and (8) other events or losses. During the post-pandemic assessment, participants were asked if they had experienced any of these events in the past year (within the last 12 months). Each event was scored as one point for its presence and zero for its absence. The total number of events was calculated by summing the number of affirmative answers.
- Brief Resilience Scale (BRS) [71]. The BRS is a six-item scale that assess “resilience as the ability to bounce back or recover from stress” [71] (p. 194). The response scale is five points. It ranges from 1 (strongly disagree) to 5 (strongly agree). Cronbach’s alpha coefficient was 0.81 for the current sample.
- Perceived Vulnerability to Disease Questionnaire (PVD) [72] in the Spanish translation by Díaz et al. [73]. PVD is a 15-item self-report instrument that assesses beliefs about personal susceptibility to infectious disease transmission and emotional discomfort in the presence of potential disease transmission. It has been used in several countries, and data from young adults in 16 countries during the pandemic revealed a global factor associated with the fear of COVID-19 across various levels of threat [74]. The response scale is a seven-point Likert scale ranging from 1 (strongly disagree) to 7 (strongly agree). The Cronbach’s alpha coefficient for the 15 items was 0.78 for the current sample.

The following sociodemographic variables have been measured: gender (women, men, other), age, educational level, occupation, number of children, and marital status. Very few people responded to the gender category “other,” so these individuals were excluded from the study.

### 2.3. Procedure

Data were collected via an online survey using a Google Form. After obtaining informed consent, the measures described in the Instruments subsection were presented. All participants were volunteers and received no material incentives for participating. Access to the sample was through the researchers’ social network. It was also through the social network of undergraduate and graduate students enrolled at various Spanish universities. These students received course credits for their participation in data collection. This study is part of a broader investigation into the impact of the pandemic in Spain. Participants were included in this study if they met the following criteria: (1) Being between 30 and 59 years of age. (2) Women and men of similar ages. (3) Not being a student. The study complied with the 1964 Helsinki Declaration and subsequent amendments, and no names or identifying information were used in the survey. The Ethics Committee for Human Research (CEIH) of the University of Seville’s Pablo de Olavide and the Ethics of Research and Animal Well-Being Committee of the University of La Laguna positively evaluated the research.

### 2.4. Data Analysis

Descriptive analyses were conducted to examine the distribution of the variables and to analyze the demographic characteristics of the participants. Cronbach’s alpha coefficient was used to calculate internal consistency. Analyses of variance (ANOVA) were performed to achieve the first objective of the study, which was to know if there were differences in psychological distress, stress, social support, and well-being by gender and pandemic period (lockdown, second and third pandemic waves, last year of the pandemic and post-pandemic) among Spanish adults at two stages of this life cycle: established adulthood and midlife. In all ANOVAs, the factors were gender (men, women) and phase of the pandemic (lockdown, second and third pandemic waves, last year of the pandemic, and post-pandemic), and the dependent variables were psychological distress in the first analysis, negative feelings, positive feelings, affect balance, thriving, life satisfaction, self-esteem, emotional social support, instrumental social support, and number of stressful events. These analyses were conducted independently in the two age groups. To determine which groups showed statistically significant differences, post hoc analyses were performed using the Scheffé adjustment when the ANOVA assumption of homogeneity of variance was met. When this assumption was not met, i.e., when the variance of the groups in the dependent variable was not equal, the nonparametric Games–Howell test was used.

To address the second aim of the study, which was to identify the main predictors of psychological distress and well-being in women and men in each age group in the post-pandemic period, hierarchical multiple regression analyses were conducted. Well-being scores were calculated by summing the scores for affect balance, thriving, and life satisfaction. In the first step of each analysis (Model 1), demographic variables (age, number of children, education level, and presence of a partner) were included. The number of children and the age of the respondents were introduced as quantitative variables. The education level was included as an ordinal variable with four levels, ranging from 0 (no formal education) to 4 (university education). The presence or absence of a partner was included as a dummy variable with two levels: no partner, value 0; partner, value 1. In step 2 (Model 2), perceived vulnerability to infection was added. In Step 3 (Model 3), the number of stressful events was added. In step 4 (Model 4), stress resilience was added. In Step 5 (Model 5), scores for self-esteem, emotional social support, and instrumental social support were added. Statistical analyses were performed using IBM SPSS Statistics 29.0.

## 3. Results

### 3.1. Differences in Psychological Distress, Well-Being, Social Support, and Stress by Gender and Pandemic Period

Table 2 shows the main results of the two-way ANOVA statistics for study variables for the established adulthood group (ages 30–44), and Table 3 shows the results for the midlife group (ages 45–59). As these tables show, when psychological distress score was the dependent variable, the gender × pandemic period interaction was not statistically significant, though the main effects of gender and pandemic period were. The effect size was small, particularly for the effect of the pandemic period. The Games–Howell test was used to determine which groups showed statistically significant differences, as Levene’s test for homogeneity of variance was statistically significant. Games–Howell Post hoc test revealed statistically significant differences between women and men in the established adulthood group during the second and third waves of the pandemic (*p* = 0.007), during the last year of the pandemic (*p* = 0.018), and during the post-pandemic period (*p* < 0.001). Women experienced greater psychological distress than men during these periods. Furthermore, women experienced greater psychological distress during the second and third waves of the pandemic than during the lockdown (*p* = 0.015). Midlife women experienced higher levels of psychological distress than men during the second and third waves of the pandemic (*p* = 0.001), during the last year of the pandemic (*p* = 0.024), and during the post-pandemic period (*p* = 0.002). Additionally, women in this age group experienced higher levels of psychological distress during the second and third waves of the pandemic than during the final year of the pandemic (*p* = 0.005) and the post-pandemic period (*p* < 0.001).

During the lockdown period, the psychological distress rate for men in established adulthood was 35.5%. It was 46.8% during the second and third waves, 32.7% during the last year of the pandemic, and 28.6% during the post-pandemic period. Among midlife men, the rates were 38.5%, 38.0%, 24.1%, and 22.0%, respectively. For women in established adulthood, the rates were 49.7% during lockdown, 61.1% during the second and third waves, 49.6% during the last year of the pandemic, and 47.5% during the post-pandemic period. Among midlife women, the rates were 52.7%, 56.6%, 40.4%, and 34.7%, respectively. The differences in these rates according to life stage were not statistically significant for men but were significant for women during the last year of the pandemic and the post-pandemic period (*p* = 0.02), with the highest rates in the youngest group of women.

**Figure 1:**
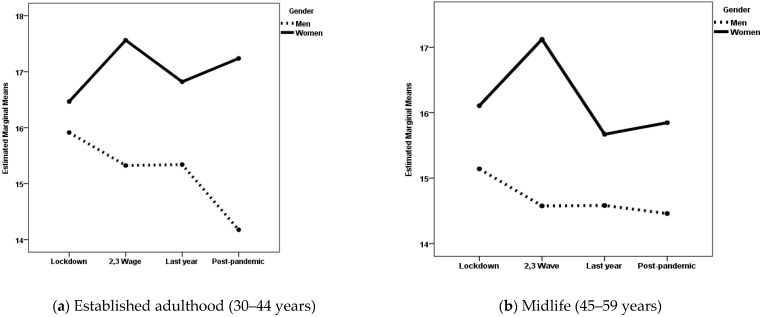
Estimated marginal means of negative feelings experienced by women and men during the four periods of the COVID-19 pandemic.

When negative feelings were the dependent variable, the gender × pandemic period interaction was statistically significant for both age groups. The effect size of the interaction was small for the established adulthood group and very small for the midlife group. The main effects of gender were also statistically significant for both groups, with a small effect size. The main effect of the pandemic period was statistically significant for the midlife group, although the size effect was very small. Figure 1a displays the estimated marginal means of negative feelings experienced by women and men during the four periods of the COVID-19 pandemic, while Figure 1b displays the same information for the midlife group. Post hoc analyses with Scheffé adjustment revealed statistically significant differences between women and men for both groups during the second and third pandemic waves (*p* < 0.001) and in the post-pandemic period. In both pandemic periods, women scored higher than men. However, during the post-pandemic period, the magnitude of the gender differences was smaller among the midlife group than the established adulthood group. The average score was 14.46 for men and 15.85 for women in the midlife group (*p* = 0.028). For the established adulthood group, the average scores were 14.18 and 17.24 for men and women, respectively (*p* < 0.001). In addition, there were also statistically significant differences among women in midlife depending on the pandemic period, with higher scores during the second and third waves than during the last year of the pandemic (*p* = 0.005) and the post-pandemic period (*p* < 0.001).

When positive feelings were the dependent variable, neither the gender × pandemic period interaction nor the main effect of gender was statistically significant in any life cycle period. However, the main effect of the pandemic period was statistically significant for both groups, with a small effect size. Post hoc analyses with Scheffé adjustment showed that statistically significant differences (*p* < 0.001) were only found in midlife women, with more positive feelings during the last year of the pandemic and the post-pandemic period than during the second and third pandemic waves.

**Figure 2:**
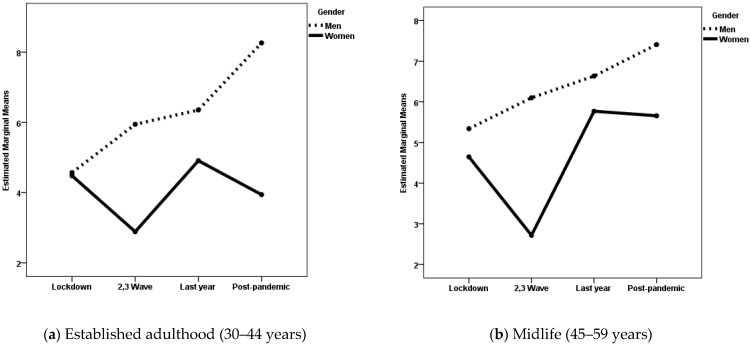
Estimated marginal means of affect balance for women and men during the four periods of the COVID-19 pandemic.

When the dependent variable was affect balance, the gender × pandemic period interaction was statistically significant in both life cycle stages. The effect size was small for the established adulthood group and very small for the midlife group. The main effects of gender and pandemic period were also statistically significant for both groups, with small effect sizes. Figure 2a shows the estimated marginal means for women and men during the four periods of the COVID-19 pandemic for the established adulthood group, and Figure 2b shows the same for the midlife group. Post hoc analyses with Scheffé adjustment revealed statistically significant differences between women and men during the second and third pandemic waves for the established adulthood group (*p* = 0.032) and for the midlife group (*p* < 0.001). Additionally, there were statistically significant differences (*p* < 0.001) in the post-pandemic period for the established adulthood group. Women had lower affect balance scores than men. Among midlife women, there were also statistically significant differences as a function of pandemic period. They had greater affect balance during the last year of the pandemic and the post-pandemic period than during the second and third pandemic waves (*p* < 0.001).

When the dependent variable was thriving, the gender × pandemic period interaction was not statistically significant in either life cycle period. The main effect of gender was statistically significant for the established adulthood group, although the effect size was negligible. The main effect of the pandemic period was significant for the midlife group, with a small effect size. Post hoc analyses with Scheffé adjustment revealed statistically significant differences only among women in the midlife stage. Relative to the last year of the pandemic (*p* = 0.037) and the post-pandemic period (*p* = 0.016), midlife women experienced lower levels of thriving during the second and third pandemic waves.

**Figure 3:**
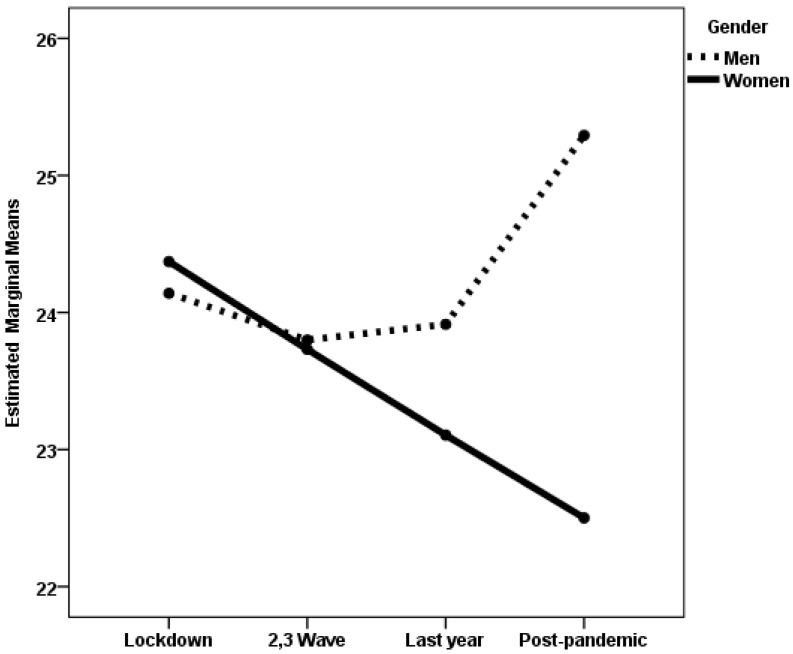
Estimated marginal means of Life satisfaction experienced by established adulthood women and men during the four periods of the COVID-19 pandemic.

When life satisfaction was the dependent variable, the gender × pandemic period interaction was statistically significant in the established adulthood group. The effect size was very small. This group also showed a statistically significant main effect of gender, albeit with a negligible effect size. Figure 3 shows the estimated marginal means for women and men during the four periods of the COVID-19 pandemic at this life stage. Post hoc analyses with Scheffé adjustment revealed statistically significant differences between women and men only during the post-pandemic period (*p* < 0.001), with women reporting lower life satisfaction than men. In the midlife group, although the main effect of the pandemic period was statistically significant, the effect size was very small. Post hoc analyses with Scheffé adjustment did not reveal any statistically significant differences.

When self-esteem was the dependent variable, the gender × pandemic period interaction was not statistically significant in either group. The main effect of gender was statistically significant in the established adulthood group and the main effect of the pandemic period in the midlife group, although the effect sizes were very small for both groups. Post hoc analyses with Scheffé adjustment revealed no statistically significant differences between the groups.

**Figure 4:**
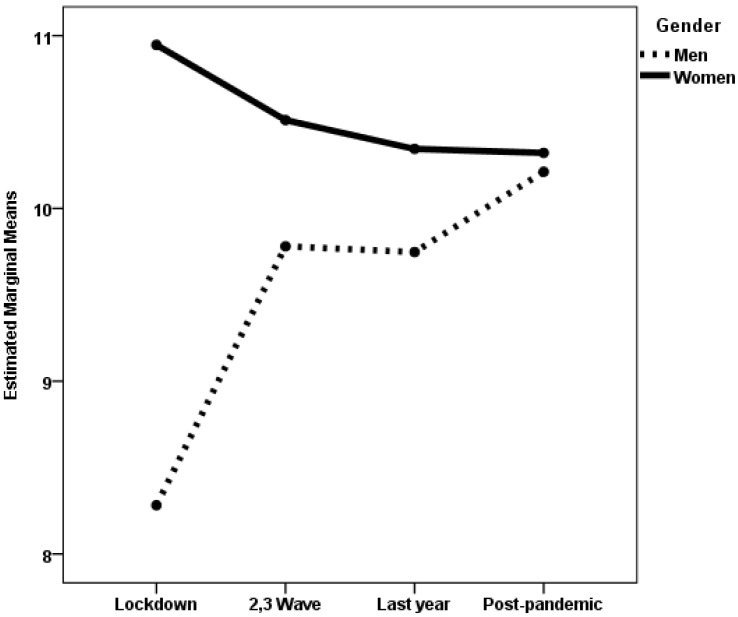
Estimated marginal means of instrumental social support perceived by established adulthood women and men during the four periods of the COVID-19 pandemic.

When emotional social support was the dependent variable, no statistically significant effect was found in any of the groups. When instrumental social support was the dependent variable, the interaction gender × pandemic period was statistically significant in the established adulthood group. The effect size was small. Furthermore, during this life stage, the main effect of gender was also statistically significant, with a small effect size. Post hoc analyses with Scheffé adjustment revealed statistically significant gender differences (*p* < 0.001) only during the lockdown period. During this time, perceived instrumental social support was higher for women than for men, as illustrated in Figure 4. Furthermore, men perceived less instrumental support during the lockdown than in the post-pandemic period (*p* = 0.049). In the midlife group, the main effects of gender and pandemic period were statistically significant, though the effect sizes were very small (see Table 3). Post hoc analyses with Scheffé adjustment revealed no statistically significant differences for this group.

**Figure 5:**
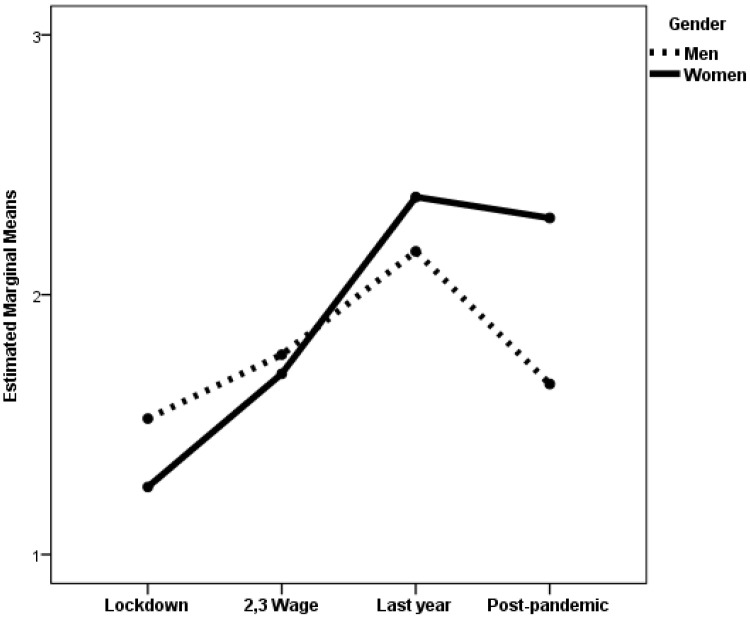
Estimated marginal means of the number of stressful events experienced by established adulthood women and men during the four periods of the COVID-19 pandemic.

When the dependent variable was the number of stressful events, the gender × pandemic period interaction was statistically significant in the established adulthood group (see Figure 5). The effect size was small. This group also showed a statistically significant main effect of the pandemic period, with a small effect size. Post hoc analyses with a Games–Howell adjustment revealed that, during the post-pandemic period, women reported a higher number of stressful events than men (*p* = 0.001). Additionally, women reported fewer stressful events during lockdown than during the second and third pandemic waves (*p* = 0.029), as well as during the last year of the pandemic and the post-pandemic period (*p* < 0.001). They also reported fewer stressful events during the second and third waves than during the last year of the pandemic and the post-pandemic period (*p* < 0.001). No statistically significant differences were found between any of the pandemic periods for the group of men. In the midlife group, the main effects of gender and pandemic period were statistically significant, although the effect size was small for period and negligible for gender. Post hoc analyses with Scheffé adjustment showed no statistically significant differences in the number of stressful events between women and men, though both genders reported fewer stressful events during the lockdown and second and third pandemic waves than during the last year of the pandemic (*p* < 0.05). Additionally, women reported fewer stressful events during the lockdown and second and third pandemic waves than during the post-pandemic period (*p* < 0.05).

Table 4 shows the stressful events experienced in the past year by women and men in both life stages during the post-pandemic period. In both groups, women reported major disagreements with family members than men did. Furthermore, in the established adulthood group, women reported major disagreements with their partners and their own illnesses more frequently than men did. Midlife women reported illnesses of family members and/or loved ones, as well as financial problems, more frequently than men did. The most frequently reported event across all groups was the illness of family members or loved ones, mentioned by half of the participants. This was followed by the death of one or more family members or loved ones, which a third of the sample reported. For the established adulthood group, the next most frequently cited event was major disagreements with their partner. For the midlife group, it was one’s own illness. Financial problems were cited by 25% of the participants, except for midlife men, of whom only 16.6% mentioned this issue. Loss of employment was more prevalent in the established adulthood group, where it was reported by 16.6% of men and 13.9% of women, compared to fewer than 10% in the midlife group. Although it was a minority, women were more likely than men to report that other stressful events had occurred in the last year. While the types of events cited varied, it was most common for participants to cite specific physical or mental illnesses of their own or of family members.

### 3.2. Predictors of Psychological Distress and Well-Being Among Women and Men at Each Life Stage During the Post-Pandemic Period

Table 5 displays the main results of the hierarchical regression analyses predicting psychological distress for the two groups of women. Table 6 shows the results for men. The sociodemographic variables included in Model 1 did not produce statistically significant changes in R^2^ in any of the groups except for midlife men. The only statistically significant Beta weight for this group was that of married/partnered, which also occurred in the group of men in established adulthood. Married/partnered men experienced lower psychological distress. Addition of perceived vulnerability to infection to the equation in Model 2 resulted in significant increments in R^2^ in all groups except among men in established adulthood. Higher psychological distress was associated with greater perceived vulnerability to infection. Addition of the number of stressful events to the equation in Model 3 resulted in significant increment in R^2^ in all groups. People who reported a higher number of stressful events during the previous year experienced greater psychological distress. Adding stress resilience to the regression (Model 4) generated a significant increment in R^2^ in all groups, indicating greater psychological distress in individuals with lower stress resilience. Adding self-esteem and emotional and instrumental social support to Model 5 generated a statistically significant increment in R^2^ in all groups. The Beta weight of self-esteem was statistically significant in all groups. People with higher self-esteem tend to experience lower psychological distress. Additionally, among established adult men, the beta weight of emotional social support was statistically significant. For midlife men, the beta weight of instrumental social support was statistically significant. Men with greater perceived social support experienced lower levels of psychological distress.

In the final model, in which all variables were included in the regression equation, self-esteem was the main predictor of psychological distress for all four groups. For both women’s groups, stress resilience and the number of stressful events experienced in the past year were statistically significant predictors. Furthermore, for midlife women, perceived vulnerability to infection was another statistically significant predictor. The percentage of variance predicted was 47% for established adulthood women and 51% for midlife women. For established adulthood men, the second most relevant predictor of psychological distress was emotional social support. Being married or living with a partner and the number of stressful events during the previous year were the third and fourth most relevant predictors, respectively. The percentage of variance predicted was 48%. For midlife men, the second most important predictor of psychological distress was the number of stressful events in the previous year, followed by instrumental social support and stress resilience. The percentage of variance predicted for this group of men was 38%.

The results of the hierarchical regression analyses predicting well-being in the two groups of women are shown in Table 7, and those of the men are shown in Table 8. As can be seen, the sociodemographic variables included in Model 1 produced statistically significant changes in R^2^ in all groups except among men in established adulthood. For both groups of women, the sociodemographic variables with statistically significant Beta weights were the number of children and education. Women with more children and a higher level of education reported greater well-being. In contrast, for men in midlife, the statistically significant Beta weight was married/partnered, with greater well-being observed in married or partnered men. In all groups except for men in established adulthood, the addition of perceived vulnerability to infection in Model 2 resulted in a statistically significant increment in R^2^: lower well-being was reported by women and midlife men with higher perceived vulnerability to infection. Addition of the number of stressful events to the equation in Model 3 resulted in a significant increment in R^2^ across all groups. Those who reported a greater number of stressful events during the previous year showed lower well-being. Addition of stress resilience to the regression equation (Model 4) resulted in large, statistically significant increment in R^2^ in all groups, indicating higher well-being among those with higher stress resilience. Addition of self-esteem and emotional and instrumental social support in Model 5 also resulted in an important and statistically significant increment in R^2^ in all groups, with greater well-being in people with higher self-esteem. The beta weight of emotional social support was statistically significant for both groups of women, with greater well-being in women who perceived more emotional social support. Among midlife men, the beta weights of instrumental and emotional social support were also statistically significant, indicating greater well-being among those with higher levels of both types of social support.

In the final model, with all variables in the regression equation, higher self-esteem was the main predictor variable for greater well-being. For all groups except established adulthood women, greater stress resilience was the second most important predictor variable. Among men in established adulthood, only the beta weights of these two variables were statistically significant, and the percentage of variance predicted was 65%. For women in established adulthood, higher emotional social support was the second most important predictor for greater well-being, followed by greater stress resilience and a lower number of stressful life events in the previous year. The percentage of variance predicted for this group was 66%. For midlife women, the third most relevant predictor of greater well-being was greater perceived emotional support, followed by experiencing a lower number of stressful life events during the previous year, and greater number of children. The percentage of variance predicted for this group was 63%. For midlife men, the other statistically significant predictors of greater well-being were experiencing a lower number of stressful life events during the previous year and perceiving greater emotional and instrumental social support. The percentage of variance predicted for this group was 58%.

## 4. Discussion

This study examined differences in psychological distress, stress, social support, and well-being according to gender and pandemic period during four periods (lockdown, second and third pandemic waves, last year of the pandemic, and post-pandemic) of the COVID-19 pandemic, which involved different risks and situations for the population. The analysis focused on the Spanish population during two life stages that present different career, familial, and physical challenges for most people [48]: established adulthood (ages 30 to 44) and midlife (ages 45 to 59). The results showed that the pandemic significantly impacted mental health, as found in other studies [5,6,15,18,23,24,30,75], but this risk varied depending on gender, pandemic period, life stage, and health indicator.

Psychological distress was one of the most widely studied variables worldwide during the COVID-19 pandemic [5,6,23,25,26,27,32,38,39,47,53,76]. Most studies have found that psychological distress increased during the onset of the pandemic and that distress rates were higher for women than for men [5,6,25,32,38,39,75,76]. While the current study does not include measures of psychological distress prior to the pandemic and cannot address variations relative to the pre-pandemic period, it has been found that rates of psychological distress have been higher for women than for men in all analyzed periods. Furthermore, rates of psychological distress differ depending on the analyzed pandemic period. These results are consistent with findings from other countries, where it has been observed that rates of psychological distress vary depending on the pandemic situation [25,26,27,77]. Findings of this study suggest that life stage (established adulthood versus midlife) is not a source of statistically significant differences in psychological distress rates among men. However, higher rates were observed among women in established adulthood compared to those in midlife during the last year of the pandemic and in the post-pandemic period. According to studies conducted in other countries during the early years of the pandemic, younger people are at greater risk of mental health problems compared to older people [5,11,28,29,30,31,32,77]. The findings of this study confirm that the risk of psychological distress remains higher for younger women than for older women at the end of the pandemic and during the post-pandemic period.

While greater psychological distress among women than men is a common finding [5,6,25,32,38,39,75,76,77], the results of this study highlight that the differences in psychological distress means between women and men were not statistically significant during the lockdown period, though they were in other pandemic periods as well as during the post-pandemic period. There were also no statistically significant differences between women and men in negative feelings or affect balance during lockdown, although there were statistically significant differences during the second and third waves of the pandemic. These results suggest that the second and third waves of the pandemic posed a higher risk of psychological distress for women than men. Although the reasons for this are unknown; it could be because the lockdown situation of women and men was more similar, as neither could leave their homes, something that did not happen during the second and third pandemic waves. Furthermore, women in both age groups reported more negative feelings than men during the post-pandemic period. These results suggest that the COVID-19 pandemic posed a greater risk to women’s mental health than to men’s, and that this increased risk persisted after the pandemic ended. These results contribute to the growing international body of evidence indicating that the COVID-19 pandemic exacerbated the gender inequalities faced by women [18,78,79,80].

When analyzing stressful events during the post-pandemic period, it was found that the most frequently cited event in all groups was the illness of family members or loved ones. Around half of the sample cited this event, followed by the death of one or more relatives or loved ones, cited by one-third of the sample. These results suggest that although the global health emergency of the pandemic has ended, health risks remain, as experts and official international organizations have pointed out [49,58]. The analysis of stressful events during the last year revealed that women in both age groups reported major disagreements with family more often than men. This may be a consequence of the pressure placed on women by the widespread expectation that women are responsible for the family [49,50,51]. Perhaps this pressure is greater for younger women, since the results of this study showed that major disagreements with family was more prevalent among younger women (27.3%) than older women (19.2%). These results are consistent with the perspective that caring for young children is one challenge of established adulthood [48], which could be a source of stress. However, the results of this study suggest that this is more stressful for women than for men. This finding aligns with international and national studies that have shown that women devote more time to family care than men do [49,50,51,52]. Additionally, women in established adulthood were more likely than men to report major disagreement with their partner (34% of women and 18.4% of men), which is consistent with the view that one of the challenges of established adulthood is maintaining relationships [48], although this seems to be a greater source of stress for women than for men. More than a fifth of participants reported their own illness, except for men in established adulthood, who reported it in only 8.6% of cases. These results are consistent with the view that people in established adulthood have fewer health risks than those in midlife [48], although this lower risk appears to be limited to men in the case of Spanish people. Financial problems were also quite common, affecting at least a quarter of the sample, except among midlife men, who cited them in only 16.6% of cases. This may reflect the economic discrimination that women generally face worldwide [50], as well as the greater job instability and economic burdens experienced by younger people compared to older people.

The second aim of the study was to identify the risk and protective factors for psychological distress and well-being among women and men in each life cycle group during the post-pandemic period. Regression analyses revealed that, among men, marital status emerged as the only relevant sociodemographic variable, with lower psychological distress among those who were married or had a partner. Notably, among men in the established adulthood group, being married or living with a partner was the third most important protective factor against psychological distress after high self-esteem and emotional social support. However, for midlife men, the beta weight of being married or living with a partner ceased to be statistically significant in the final model when self-esteem and social support were included. Although previous international studies have found that being married or living with a partner can be beneficial for mental health [6,19,32,34,35], the results of this study suggest that this only applies to men, particularly to those in established adulthood, and not to women.

Lower self-esteem and experiencing a greater number of stressful events in the previous year were important predictors of higher psychological distress for all groups studied. These results are consistent with previous research conducted in other countries [41,42]. Moreover, for all groups except for established adulthood men, having lower stress resilience was another relevant predictor. For the two women’s groups, stress resilience and experiencing fewer stressful events were important protective factors against psychological distress. For midlife men, however, experiencing fewer stressful events was more relevant than having greater stress resilience. Perceived vulnerability to infection was a risk factor for greater psychological distress in all groups except for men in established adulthood. However, except among midlife women, this variable ceased to be statistically significant when self-esteem and social support were included in the regression equation.

For all groups, high self-esteem was the main predictor of greater well-being and the leading protective factor against psychological distress. These results align with previous research [38,39,41,42,76] suggesting the significant role of self-esteem in population mental health. High stress resilience was also a relevant factor for well-being in all groups. Furthermore, it was a significant predictor of lower psychological distress for all groups except among established adult men. These results are consistent with previous research [31,32,37,40,76] that identified resilience as an important factor for mental health. Another relevant predictor of well-being was experiencing fewer stressful life events. However, among established adult men, this factor was not statistically significant in the final model that included self-esteem and social support. Previous international research [35,36,37,38,39] has shown that social support is a protective factor for mental health during the COVID-19 pandemic. However, the results of the present study found that, during the post-pandemic period, the protective role of social support against psychological distress appear to be limited to men. For women, greater perceived emotional social support is associated with greater well-being. Social support is not associated with well-being in men during established adulthood. However, a greater perception of emotional and instrumental social support is associated with greater well-being among midlife men.

### Limitations and Future Research

The results of this study contribute to our understanding of the impact of different periods of the pandemic and gender on mental health. They add to the existing evidence that the pandemic posed a greater risk to women’s mental health than to men’s, as observed in previous research [5,6,20,28,29,30,31,32,78,79,80]. However, when interpreting the results, the limitations of the study must be considered. First, this is a cross-sectional study. This means that causal relationships cannot be established. The differences observed across pandemic phases may reflect variations between independent cohorts rather than within-person changes over time. Moreover, although self-esteem and resilience were identified as key predictors of psychological distress and well-being, causality cannot be inferred. It is equally plausible that psychological distress erodes self-esteem and resilience, rather than the reverse. Future research should employ prospective longitudinal designs so that causal attributions can be established. The sample is not probabilistic, which limits the generalization of the results. Recruitment through social networks and university contacts likely favored more educated, urban, and digitally connected participants. This may result in an underrepresentation of vulnerable groups, such as individuals with lower socioeconomic status, rural populations, and older adults. Additionally, the study excluded individuals who identified as “other genders” due to the small number of respondents in this category, which poses challenges for statistical analyses and precludes robust conclusions. Future studies should use probabilistic samples and be more inclusive of people of other genders. The number of people in the different pandemic periods studied varies. This may affect the ability to detect statistically significant differences.

All measures are self-reported. Sole reliance on self-administered questionnaires introduces potential reporting biases, which could contribute to the observed gender differences (e.g., women may report symptoms more readily than men). In addition, the specific self-report instruments used may also generate biases in the results found, although attempts have been made to mitigate such effects by using validated instruments. Future research should supplement self-administered questionnaires with clinical-related or psychological assessments to validate self-reported data. Moreover, findings are situated within Spain, which had a distinctive pandemic trajectory and policy response. This limits generalizability to other cultural or policy contexts.

Future research should also explore other age groups, different cultural contexts, and the impact of specific interventions for promoting self-esteem and resilience, given their evidence as key protective factors in the current study.

## 5. Conclusions

The results of this study suggest that the threat to mental health posed by the COVID-19 pandemic was greater for women than for men, and this threat persists in the post-pandemic period, notably among women in established adulthood. In the post-pandemic period, self-esteem was the most important protective factor for mental health and well-being, followed by high resilience to stress. For all groups except for men in established adulthood, experiencing fewer stressful events was also important. The results of this study are relevant for the design of policies, programs, and strategies to achieve better health and well-being among citizens and greater gender equality. Although self-esteem is a key variable in the design of intervention programs, the results of this study highlight the importance of taking a gender and life cycle approach in the design of intervention programs to improve mental health, in prevention programs, and in the design of public policies to achieve greater health and well-being among citizens and greater gender equality.

## Figures and Tables

**Table 1:** Sociodemographic characteristics of participants.

	Men		Women		χ^2^	*p*
Variables	*n*	%	*n*	%		
Level of Education:					24.47	<0.001
Primary	279	22.5	464	19.2		
Secondary	440	35.5	732	30.2		
University	520	42.0	1225	50.6		
Non-data	5		12			
Occupation:					59.18	<0.001
Employed	1094	88.9	1890	78.6		
Unemployed	104	8.4	377	15.7		
Other	33	2.7	139	5.8		
Non-data	13		27			
Marital status:					9.68	0.008
Never married	263	21.3	501	20.7		
Married/partnered	824	66.6	1532	63.3		
Separated/divorced/widowed	150	12.1	386	16.0		
Non-data	7		14			
	M	SD	M	SD	t	p
Age	45.73	8.52	45.71	8.25	0.04	0.97
Number of children	1.26	1.10	1.32	0.99	−1.70	0.09

**Table 2:** Means, standard deviations, and two-way ANOVA statistics for study variables for the established adulthood group.

Variable	Men		Women		ANOVA		
	M	SD	M	SD	Effect	F Ratio	η_p_^2^
*Psychological distress*							
Lockdown	13.09	6.68	**14.22**	6.84			
Second and Third pandemic waves	**13.84**	6.69	**16.32**	6.71	**Period**	4.34 ***	**0.009**
Last year of the pandemic	**12.73**	5.81	**15.24**	7.51	**Gender**	38.00 ***	**0.025**
Post-pandemic period	**11.83**	5.74	**15.08**	7.02	Period × Gender	1.21	0.002
*Negative feelings*							
Lockdown	15.91	4.51	16.47	4.79			
Second and Third pandemic waves	**15.32**	4.34	**17.56**	4.39	Period	1.90	0.004
Last year of the pandemic	15.34	4.12	16.82	4.50	**Gender**	55.34 ***	**0.036**
Post-pandemic period	**14.18**	4.06	**17.24**	4.26	**Period × Gender**	4.53 **	**0.009**
*Positive feelings*							
Lockdown	20.48	4.15	20.95	4.32			
Second and Third pandemic waves	21.27	4.31	20.45	3.99	**Period**	5.66 **	**0.011**
Last year of the pandemic	21.69	4.62	21.73	4.34	Gender	2.71	0.002
Post-pandemic period	22.44	4.07	21.18	4.55	Period × Gender	2.61	0.005
*Affect Balance*							
Lockdown	4.57	7.71	4.48	8.29			
Second and Third pandemic waves	**5.94**	7.59	**2.89**	7.51	**Period**	4.02 **	**0.008**
Last year of the pandemic	6.35	7.82	4.91	7.99	**Gender**	26.03 ***	**0.017**
Post-pandemic period	**8.26**	7.24	**3.94**	7.96	**Period × Gender**	4.33 **	**0.009**
*Thriving*							
Lockdown	37.14	6.51	37.77	6.54			
Second and Third pandemic waves	37.81	7.34	36.54	6.54	Period	1.54	0.003
Last year of the pandemic	38.59	6.56	37.61	7.23	**Gender**	4.99 *	**0.003**
Post-pandemic period	38.96	6.58	37.17	7.30	Period × Gender	1.66	0.003
*Life satisfaction*							
Lockdown	24.14	7.05	24.37	6.35			
Second and Third pandemic waves	23.80	6.74	23.73	6.71	Period	0.56	0.001
Last year of the pandemic	23.91	6.64	23.10	6.98	**Gender**	5.07 *	**0.003**
Post-pandemic period	**25.29**	6.36	**22.50**	7.25	**Period × Gender**	3.57 *	**0.007**
*Self-esteem*							
Lockdown	20.97	4.76	20.79	4.92			
Second and Third pandemic waves	20.96	4.98	20.22	4.86	Period	0.62	0.001
Last year of the pandemic	21.19	5.26	20.60	5.37	**Gender**	7.48 **	**0.005**
Post-pandemic period	21.91	5.05	20.24	5.59	Period × Gender	1.24	0.003
*Emotional Social Support*							
Lockdown	15.33	4.08	16.31	4.76			
Second and Third pandemic waves	16.23	5.08	16.23	4.96	Period	1.63	0.003
Last year of the pandemic	15.49	4.33	15.89	5.04	Gender	1.38	0.001
Post-pandemic period	16.47	4.52	16.35	4.65	Period × Gender	0.77	0.002
*Instrumental Social Support*							
Lockdown	**8.28**	3.79	**10.95**	3.83			
Second and Third pandemic waves	9.78	3.89	10.51	3.97	Period	1.44	0.003
Last year of the pandemic	9.75	3.60	10.34	4.21	**Gender**	21.38 ***	**0.014**
Post-pandemic period	**10.21**	3.78	10.32	4.05	**Period × Gender**	5.49 **	**0.011**
*Number of stressful events*							
Lockdown	1.52	1.51	**1.26**	1.26			
Second and Third pandemic waves	1.77	1.40	**1.70**	1.53	**Period**	14.84 ***	0.030
Last year of the pandemic	2.17	1.89	**2.38**	1.80	Gender	2.04	0.002
Post-pandemic period	**1.66**	1.39	**2.29**	1.57	**Period × Gender**	5.12 **	0.011

Note: * *p* < 0.05; ** *p* < 0.01; *** *p* < 0.001. Statistically significant results are highlighted in bold.

**Table 3:** Means, standard deviations, and two-way ANOVA statistics for study variables for the midlife group.

Variable	Men		Women		ANOVA		
	M	SD	M	SD	Effect	F Ratio	η_p_^2^
*Psychological distress*							
Lockdown	12.53	5.89	14.61	7.09			
Second and Third pandemic waves	**13.10**	6.10	**15.48**	6.83	**Period**	9.05 ***	**0.012**
Last year of the pandemic	**12.08**	5.54	**13.81**	6.37	**Gender**	40.27 ***	**0.018**
Post-pandemic period	**11.53**	5.24	**13.32**	6.52	Period × Gender	0.29	0.000
*Negative feelings*							
Lockdown	15.14	4.83	16.11	4.63			
Second and Third pandemic waves	**14.57**	4.16	**17.12**	4.37	**Period**	3.44 *	**0.005**
Last year of the pandemic	14.58	4.51	**15.67**	4.46	**Gender**	48.23 ***	**0.022**
Post-pandemic period	**14.46**	4.05	**15.85**	4.12	**Period × Gender**	3.18 *	**0.004**
*Positive feelings*							
Lockdown	20.48	3.94	20.75	4.39			
Second and Third pandemic waves	20.67	3.88	**19.84**	4.09	**Period**	13.15 ***	**0.018**
Last year of the pandemic	21.22	4.64	**21.44**	4.20	Gender	0.74	0.000
Post-pandemic period	21.87	3.68	**21.50**	4.32	Period × Gender	1.75	0.002
*Affect Balance*							
Lockdown	5.34	7.88	4.65	8.33			
Second and Third pandemic waves	**6.10**	7.16	**2.71**	7.50	**Period**	9.03 ***	**0.012**
Last year of the pandemic	6.63	8.14	**5.77**	7.78	**Gender**	19.56 ***	**0.009**
Post-pandemic period	7.41	7.02	**5.66**	7.58	**Period × Gender**	2.90 *	**0.004**
*Thriving*							
Lockdown	38.01	5.45	37.45	6.71			
Second and Third pandemic waves	36.99	6.09	**36.03**	6.44	**Period**	8.08 ***	**0.011**
Last year of the pandemic	37.68	5.78	**37.67**	6.28	Gender	3.72	0.002
Post-pandemic period	38.72	5.96	**37.86**	6.41	Period × Gender	0.60	0.001
*Life satisfaction*							
Lockdown	23.64	6.88	23.62	6.91			
Second and Third pandemic waves	23.67	6.67	22.66	6.66	**Period**	3.55 *	**0.005**
Last year of the pandemic	24.05	6.38	23.85	6.59	Gender	2.73	0.001
Post-pandemic period	24.87	5.92	23.93	6.80	Period × Gender	0.61	0.001
*Self-esteem*							
Lockdown	21.97	4.23	21.10	4.97			
Second and Third pandemic waves	21.01	4.19	20.39	4.45	**Period**	2.76 *	**0.004**
Last year of the pandemic	21.23	4.24	21.30	5.00	Gender	3.22	0.001
Post-pandemic period	21.52	4.45	21.26	5.09	Period × Gender	0.72	0.001
*Emotional Social Support*							
Lockdown	14.82	4.61	14.79	5.06			
Second and Third pandemic waves	15.40	4.95	15.36	4.95	Period	1.27	0.002
Last year of the pandemic	14.82	5.24	15.30	5.10	Gender	0.34	0.000
Post-pandemic period	15.36	4.72	15.51	5.01	Period × Gender	0.28	0.000
*Instrumental Social Support*							
Lockdown	8.14	3.71	9.06	4.06			
Second and Third pandemic waves	8.81	4.04	9.57	4.01	**Period**	4.31 **	**0.006**
Last year of the pandemic	8.60	4.22	9.45	4.21	**Gender**	16.16 ***	**0.007**
Post-pandemic period	9.26	4.05	9.99	4.07	Period × Gender	0.04	0.000
*Number of stressful events*							
Lockdown	**1.15**	1.27	**1.46**	1.42			
Second and Third pandemic waves	**1.38**	1.48	**1.52**	1.47	**Period**	19.41 ***	**0.027**
Last year of the pandemic	**2.01**	1.65	**2.05**	1.71	**Gender**	5.58 *	**0.003**
Post-pandemic period	1.78	1.52	**2.02**	1.52	Period × Gender	0.58	0.001

Note: * *p* < 0.05; ** *p* < 0.01; *** *p* < 0.001. Statistically significant results are highlighted in bold.

**Table 4:** Stressful events experienced in the past year in the post-pandemic period by women and men in both life stages.

	Men		Women			
Event	n	%	n	%	Χ^2^	*p*
*Established adulthood (30 to 45 years)*						
Loss of employment	27	16.6	34	13.9	0.53	0.466
Financial problems	41	25.0	70	28.9	0.76	0.384
Major disagreements with partner ^a^	18	**18.4**	51	**34.0**	7.21	**0.007**
Major disagreements with family	24	**14.6**	66	**27.3**	9.05	**0.003**
Illness of family members or loved ones	80	48.8	141	58.0	3.37	0.066
Death of one or more family members or loved ones	49	29.9	86	35.4	1.34	0.247
Own illness	14	**8.6**	62	**25.5**	18.37	**<0.001**
Other events	1	**0.6**	15	**6.1**	7.98	**0.005**
*Midlife (45 to 59 years)*						
Loss of employment	29	9.6	33	9.0	0.06	0.804
Financial problems	50	**16.6**	93	**25.5**	7.92	**0.005**
Major disagreements with partner ^a^	48	20.9	36	16.3	1.56	0.212
Major disagreements with family	38	**12.6**	70	**19.2**	5.29	**0.021**
Illness of family members or loved ones	144	**47.7**	206	**56.9**	5.62	**0.018**
Death of one or more family members or loved ones	123	40.7	136	37.4	0.79	0.375
Own illness	65	21.7	101	27.9	3.93	0.065
Other events	12	4.0	32	**8.8**	6.16	**0.013**

Note: ^a^ This is only for people who have a partner. Statistically significant results are highlighted in bold.

**Table 5:** Summary of hierarchical regression results for psychological distress in established adulthood and in midlife women.

	Model 1		Model 2		Model 3		Model 4		Model 5	
Variable	β	t-Value	β	t-Value	β	t-Value	β	t-Value	β	t-Value
Established adulthood										
Age	0.01	0.11	−0.00	−0.01	−0.01	−0.18	−0.03	−0.43	−0.04	−0.73
Number of children	−0.08	−1.01	−0.03	−0.38	−0.03	−0.47	0.02	0.24	0.04	0.66
Education	**−0.14**	−2.04 *	**−0.13**	−2.02 *	−0.08	−1.22	−0.05	−0.86	−0.02	−0.45
Married/partnered	0.01	0.12	−0.04	−0.64	−0.00	−0.03	0.02	0.26	0.03	0.61
Perceived vulnerability to infection			**0.26**	3.96 ***	**0.20**	3.11 **	**0.12**	2.13 *	0.06	1.03
Number of stressful events					**0.34**	5.32 ***	**0.29**	5.06 ***	**0.19**	3.52 **
Stress resilience							**−0.42**	−7.48 ***	**−0.22**	−3.79 ***
Self-esteem									**−0.34**	5.16 ***
Emotional social support									−0.14	−1.69
Instrumental social support									−0.04	−0.45
R^2^	0.02		0.09		0.19		0.36		0.49	
Adjusted R^2^	0.00		0.07		0.17		0.34		0.47	
R^2^ Change	0.02		0.07 ***		0.11 ***		0.17 ***		0.13 ***	
Midlife women										
Age	−0.02	−0.33	−0.02	−0.45	−0.02	−0.39	−0.03	−0.76	−0.03	−0.70
Number of children	−0.07	−1.17	−0.06	−0.96	−0.06	−1.06	−0.03	−0.52	−0.01	−0.12
Education	−0.08	−1.41	−0.05	−0.95	−0.02	−0.32	0.05	1.04	0.06	1.38
Married/partnered	−0.07	−1.20	−0.08	−1.40	−0.03	−0.57	−0.05	−1.17	−0.06	−1.55
Perceived vulnerability to infection			**0.28**	5.32 ***	**0.23**	4.66 ***	**0.11**	2.45 *	**1.00**	2.49 *
Number of stressful events					**0.38**	7.82 ***	**0.34**	7.60 ***	**0.27**	6.56 ***
Stress resilience							**−0.41**	−8.94 ***	**−0.25**	−5.58 ***
Self-esteem									**−0.37**	−8.27 ***
Emotional social support									−0.11	−1.44
Instrumental social support									−0.02	−0.28
R^2^	0.02		0.09		0.23		0.38		0.52	
Adjusted R^2^	0.01		0.08		0.22		0.37		0.51	
R^2^ Change	0.02		0.08 ***		0.14 ***		0.15 ***		0.14 ***	

Note: β = standardized regression coefficient. R^2^ = explained variance. * *p* < 0.05; ** *p* < 0.01; *** *p* < 0.001. Statistically significant results are highlighted in bold.

**Table 6:** Summary of hierarchical regression results for psychological distress in established adulthood and in midlife men.

	Model 1		Model 2		Model 3		Model 4		Model 5	
Variable	β	t-Value	β	t-Value	β	t-Value	β	t-Value	β	t-Value
Established adulthood										
Age	0.02	0.22	0.02	0.22	0.05	0.52	0.05	0.64	0.05	0.77
Number of children	0.04	0.47	0.04	0.45	0.07	0.83	0.03	0.34	0.06	0.84
Education	−0.05	−0.60	−0.05	−0.55	−0.03	−0.42	−0.02	−0.34	−0.01	−0.19
Married/partnered	**−0.23**	−2.67 **	**−0.23**	−2.62 *	**−0.27**	−3.28 **	**−0.25**	−3.49 **	**−0.19**	2.82 **
Perceived vulnerability to infection			0.03	0.30	0.04	0.46	0.03	0.39	0.03	0.55
Number of stressful events					**0.32**	4.15 ***	**0.24**	3.50 **	**0.15**	2.34 *
Stress resilience							**−0.46**	6.71 ***	−0.15	−1.92
Self-esteem									**−0.45**	−5.17 ***
Emotional social support									**−0.24**	−2.24 *
Instrumental social support									0.15	1.50
*R^2^*	0.05		0.05		0.16		0.36		0.51	
Adjusted *R^2^*	0.03		0.02		0.12		0.33		0.48	
*R^2^* Change	0.05		0.00		0.10 ***		0.20 ***		0.15 ***	
Midlife										
Age	−0.02	−0.41	−0.01	−0.18	0.00	0.05	0.01	0.19	−0.01	−0.17
Number of children	0.05	0.86	0.07	1.17	0.04	0.65	0.04	0.84	0.04	0.79
Education	−0.07	−1.11	−0.05	−0.92	−0.04	−0.78	−0.02	−0.36	−0.03	−0.62
Married/partnered	**−0.18**	−3.07 **	**−0.18**	−3.10 **	**−0.15**	−2.71 **	**−0.11**	−2.20 *	−0.07	−1.39
Perceived vulnerability to infection			**0.28**	4.89 ***	**0.23**	4.20 ***	**0.12**	2.27 *	0.10	1.93
Number of stressful events					**0.32**	5.88 ***	**0.29**	5.65 ***	**0.24**	4.84 ***
Stress resilience							**−0.31**	−5.75 ***	**−0.17**	−2.99 **
Self-esteem									**−0.30**	−5.43 ***
Emotional social support									0.04	0.55
Instrumental social support									**−0.18**	−2.49 *
R^2^	0.04		0.11		0.21		0.29		0.40	
Adjusted R^2^	0.02		0.10		0.19		0.27		0.38	
R^2^ Change	0.04 *		0.07 ***		0.10 ***		0.08 ***		0.11 ***	

Note: β = standardized regression coefficient. R^2^ = explained variance. * *p* < 0.05; ** *p* < 0.01; *** *p* < 0.001. Statistically significant results are highlighted in bold.

**Table 7:** Summary of hierarchical regression results for well-being in established adulthood and in midlife women.

	Model 1		Model 2		Model 3		Model 4		Model 5	
Variable	β	t-Value	β	t-Value	β	t-Value	β	t-Value	β	t-Value
Established adulthood										
Age	−0.11	−1.61	−0.10	−1.55	−0.09	−1.46	−0.08	−1.43	−0.06	−1.48
Number of children	**0.22**	2.91 **	**0.16**	2.23 *	**0.17**	2.40 *	0.12	1.89	0.09	1.86
Education	**0.18**	2.69 **	**0.17**	2.71 **	**0.12**	2.01 *	0.09	1.74	0.06	1.37
Married/partnered	−0.01	−0.17	0.05	0.72	0.01	0.19	−0.01	−0.11	−0.03	−0.78
Perceived vulnerability to infection			**−0.30**	−4.70 ***	**−0.25**	−3.93 ***	**−0.17**	−3.00 **	−0.07	−1.65
Number of stressful events					**−0.29**	−4.70 ***	**−0.24**	−4.40 ***	**−0.10**	−2.33 *
Stress resilience							**0.45**	8.21 ***	**0.16**	3.33 **
Self-esteem									**0.50**	9.53 ***
Emotional social support									**0.22**	3.20 **
Instrumental social support									0.02	0.32
R^2^	0.06		0.15		0.23		0.41		0.68	
Adjusted R^2^	0.04		0.13		0.20		0.39		0.66	
R^2^ Change	0.06 **		0.09 ***		0.08 ***		0.18 ***		0.27 ***	
Midlife										
Age	0.03	0.56	0.03	0.64	0.03	0.60	0.05	1.06	0.04	1.18
Number of children	**0.15**	2.60 *	**0.14**	2.47 *	**0.14**	2.61 **	**0.11**	2.24 *	**0.08**	2.17*
Education	**0.15**	2.70 **	**0.13**	2.37 *	0.10	1.92	0.03	0.60	0.02	0.42
Married/partnered	0.06	1.04	0.06	1.18	0.02	0.47	0.05	1.14	0.06	1.68
Perceived vulnerability to infection			**−0.20**	−3.90 ***	**−0.16**	−3.21 **	−0.03	−0.69	−0.02	−0.51
Number of stressful events					**−0.32**	−6.35 ***	**−0.27**	−6.03 ***	**−0.16**	−4.63 ***
Stress resilience							**0.47**	10.04 ***	**0.24**	6.24 ***
Self-esteem									**0.47**	12.12 ***
Emotional social support									**0.17**	2.68 **
Instrumental social support									0.06	0.93
R^2^	0.05		0.09		0.18		0.37		0.64	
Adjusted R^2^	0.03		0.07		0.17		0.36		0.63	
R^2^ Change	0.05 **		0.04 ***		0.10 ***		0.19 ***		0.27 ***	

Note: β = standardized regression coefficient. R^2^ = explained variance. * *p* < 0.05; ** *p* < 0.01; *** *p* < 0.001. Statistically significant results are highlighted in bold.

**Table 8:** Summary of hierarchical regression results for well-being in established adulthood and in midlife men.

	Model 1		Model 2		Model 3		Model 4		Model 5	
Variable	β	t-Value	β	t-Value	β	t-Value	β	t-Value	β	t-Value
Established adulthood										
Age	0.04	0.41	0.04	0.40	0.01	0.14	0.01	0.11	0.02	0.35
Number of children	0.04	0.39	0.03	0.35	0.00	0.02	0.06	0.84	0.05	0.85
Education	0.12	1.38	0.12	1.45	0.11	1.37	0.10	1.51	0.07	1.36
Married/partnered	0.11	1.28	0.12	1.33	0.16	1.90	**0.14**	2.03 *	0.05	0.95
Perceived vulnerability to infection			0.05	0.63	0.04	0.53	0.05	0.83	0.04	0.71
Number of stressful events					**−0.32**	−4.00 ***	**−0.22**	−3.35 **	−0.07	−1.40
Stress resilience							**0.57**	8.97 ***	**0.18**	2.84 **
Self-esteem									**0.52**	7.35 ***
Emotional social support									0.07	0.83
Instrumental social support									0.15	1.77
R^2^	0.03		0.03		0.13		0.44		0.67	
Adjusted R^2^	0.01		0.00		0.10		0.42		0.65	
R^2^ Change	0.03		0.00		0.10 ***		0.31 ***		0.23 ***	
Midlife										
Age	−0.03	−0.46	−0.03	−0.60	−0.05	−0.82	−0.06	−1.11	−0.02	−0.62
Number of children	0.01	0.17	0.01	0.02	0.03	0.52	0.02	0.36	0.02	0.39
Education	0.09	1.59	0.09	1.48	0.08	1.38	0.04	0.87	0.07	1.85
Married/partnered	**0.23**	3.94 ***	**0.23**	3.93 ***	**0.20**	3.61 ***	**0.16**	3.06 **	0.06	1.36
Perceived vulnerability to infection			**−0.15**	−2.60 *	−0.11	−1.89	0.04	0.75	0.08	1.86
Number of stressful events					**−0.28**	−5.02 ***	**−0.24**	−4.80 ***	**−0.15**	−3.66 ***
Stress resilience							**0.44**	8.21 ***	**0.19**	4.06 ***
Self-esteem									**0.46**	9.96 ***
Emotional social support									**0.15**	2.31 *
Instrumental social support									**0.14**	2.40 *
R^2^	0.06		0.08		0.16		0.32		0.60	
Adjusted R^2^	0.05		0.07		0.14		0.31		0.58	
R^2^ Change	0.06 **		0.02 *		0.08 ***		0.16 ***		0.28 ***	

Note: β = standardized regression coefficient. R^2^ = explained variance. * *p* < 0.05; ** *p* < 0.01; *** *p* < 0.001. Statistically significant results are highlighted in bold.

## Data Availability

The raw data supporting the conclusions of this article will be made available by the authors on request.
